# Prognostic utility of late gadolinium enhancement cardiac magnetic resonance imaging in coronary artery disease: a meta-analysis

**DOI:** 10.1186/1532-429X-15-S1-O75

**Published:** 2013-01-30

**Authors:** Raymond H Chan, Alexander A Leung, Warren J Manning

**Affiliations:** 1 Beth Israel Deaconess Medical Center, Chestnut Hill, MA, USA; 2 Brigham and Women's Hospital, Boston, MA, USA

## Background

Late gadolinium enhancement (LGE) cardiac MR can identify injured or scarred myocardium. However its prognostic implication remains unclear.

## Objective

We sought to quantify the risk of major adverse cardiovascular events (MACE) among patients with LGE and CAD.

## Methods

Two reviewers conducted a systematic search of electronic databases (MEDLINE and EMBASE) and hand searched bibliographies. Reviewers extracted data in duplicate, evaluated the quality of the studies based on a 4 point scale, and calculated pooled estimates. Out of 579 unique records screened, 115 full-text articles were assessed for eligibility. We then performed a meta-analysis on 18 eligible studies which reported on the occurrence of MACE in patients with LGE detected after a myocardial infarction.

## Results

A total of 4,438 patients were included in the analysis. The overall hazard ratio (HR) for MACE was 2.65 (95% confidence intervals, CI, 1.98-3.56) for the presence of any LGE, with large amounts of heterogeneity between studies (I^2^, 83.5%). Furthermore, there was a continuous relationship between risk and the amount of LGE detected. For every 10% of the left ventricular mass with LGE, the risk of MACE increased by 56% (HR 1.56/10% LGE, 95% CI 1.39-1.75; I2, 63.6%). Pre-specified meta-regression analyses revealed that the HR for MACE decreased with declining ejection fraction (p=0.02) when LGE was continuous, and was inversely related to age (p<0.001) when LGE was binary.

## Limitations

Studies were heterogeneous with respect to patient characteristics and the definition of MACE, which may limit interpretability and generalizability.

## Conclusions

The presence and extent of LGE are independent predictors of MACE in patients with prior myocardial infarction.

## Funding

Nil.

**Figure 1:**
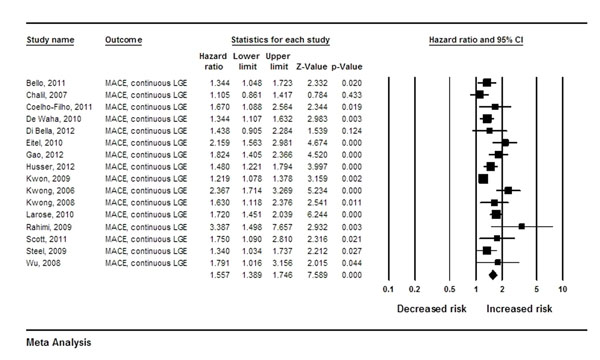
Forest Plot of risk for major adverse cardiovascular events as predicted by late gadolinium enhancement, expressed as HRs with 95% CIs.

